# Supplementary material for: Cytochrome c oxidase dependent respiration is essential for T cell activation, proliferation and memory formation
Source: Nat Commun. 2025 Dec 4;16:10898. doi: 10.1038/s41467-025-65910-w (PMC12678437; doi:10.1038/s41467-025-65910-w)
Supplement: Supplementary file 1 — Supplementary Information [file 41467_2025_65910_MOESM1_ESM.pdf]

**SUPPLEMENTARY DATA**

**Supplementary Data 1 (Excel file). Differential gene expression statistics underlying volcano plots.** Source data for Figure 1 and Supplementary Fig 1. Differential expression analyses were performed for WT vs. *TCox10*$^{-/-}$, WT vs. *Aox*, WT vs. *TCox10*$^{-/-}$*/Aox*, and *TCox10*$^{-/-}$ vs. *TCox10*$^{-/-}$*/Aox* T cells. Reported values include gene name, base mean expression, $\log_2$ fold change, test statistic, P value, and adjusted P value.

**Supplementary Data 2 (Excel file). Overrepresentation analysis of gene co-expression modules. T**able summarizes the results of overrepresentation analysis (ORA) performed on each gene module identified in the dataset for Figure 1. The analysis indicates the predominant biological functions and pathways associated with the modules. Reported values include ontology, description, BgRatio, P value, adjusted P value, Q value, gene ID, and counts. These data define the major functions represented in the gene sets underlying the modules.

**Supplementary Data 3 (Excel file). Focused transcriptomic analysis of mitochondrial pathways in *TCox10-/-/AOX* T cells.** Table contains the pathway-level transcriptomic results corresponding to Supplementary Fig. 2. Analyses were performed to determine whether *Aox* expression influences mitochondrial programs in *TCox10*$^{-/-}$ T cells. Reported values include gene ID and description, set size, enrichment score, normalized enrichment score (NES), P value, adjusted P value, Q value, rank, leading edge, core enrichment.

**Supplementary Data 4 (Excel file). Transcriptomic analysis of memory T cell differentiation in *TCox10*$^{-/-}$*/Aox* cells.** This table contains the RNAseq results corresponding to Supplementary Fig. 5B. Differential expression was assessed in *TCox10*$^{-/-}$*/Aox* memory T cells compared to WT controls. Reported values include gene name, base mean expression, $\log_2$ fold change, test statistic, P value, and adjusted P value.

## SUPPLEMENTARY FIGURES

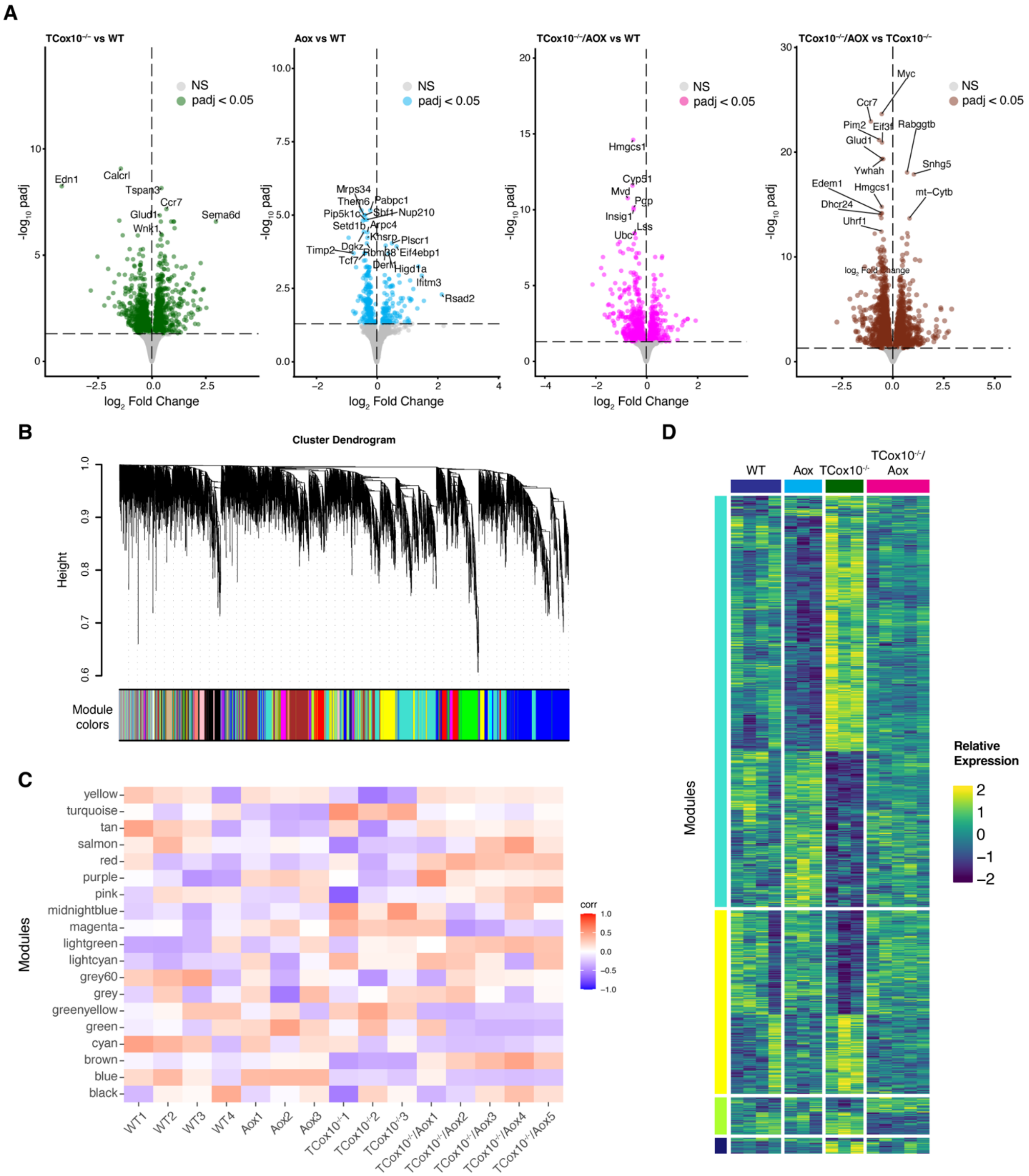


**Supplementary Fig. 1. Weighted gene correlation network analysis reveals *Aox* expression corrects aberrant *TCox10*$^{-/-}$ gene expression.** (A) Volcano plots demonstrating differential gene expression of *TCox10*$^{-/-}$, *Aox*, and *TCox10*$^{-/-}$*/Aox* T cells against WT T cells, as well as *TCox10*$^{-/-}$*/Aox* against *TCox10*$^{-/-}$. (B) Cluster dendrogram of weighted gene correlation network analysis

(WGCNA) showing distribution of genes into indicated modules. (C) Heatmap showing the correlation of eigengene intercepts across samples in the detected modules. (D) Heatmap of gene expression from the selected modules in (C). Data represents an RNAseq experiment; n = 3-4.

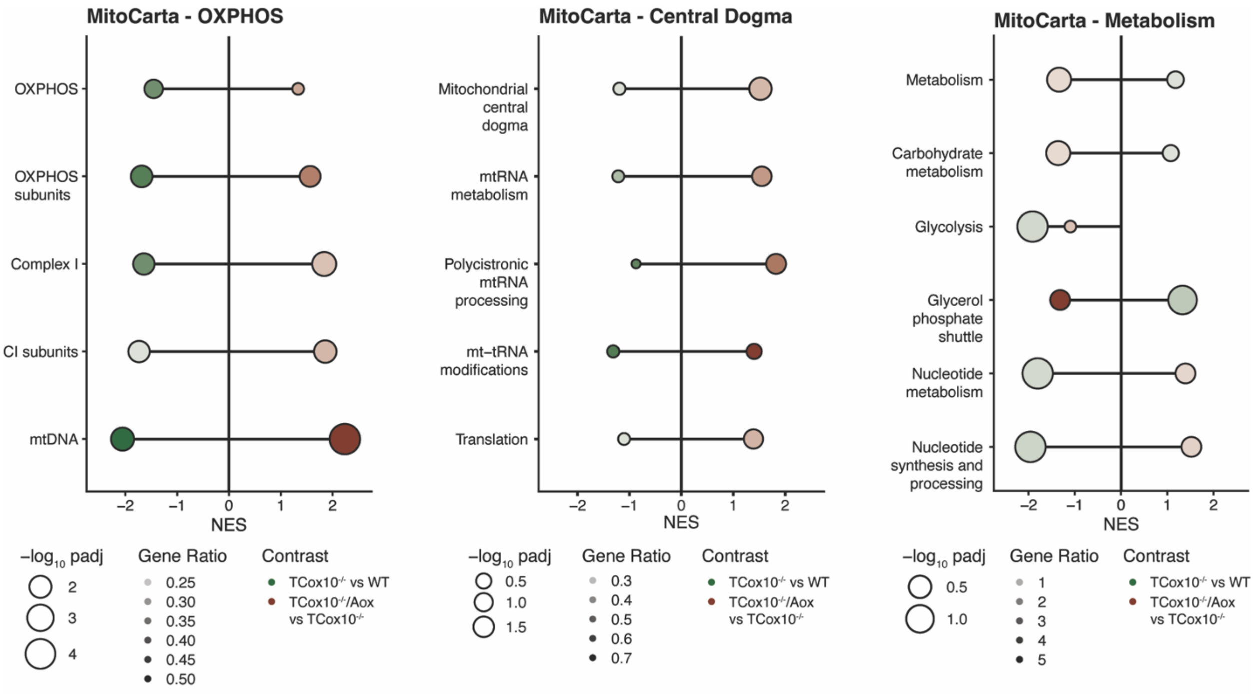


**Supplementary Fig. 2. *Aox* expression improves OXPHOS and mtRNA gene expression and mitochondrial function.** Lollipop plots of gene set enrichment analysis (GSEA) of MitoCarta pathways, separated between OXPHOS, central dogma, and metabolism categories. Point size reflects -log10 B-H adjusted p value, point opacity reflects ratio of enriched genes to genes in the set. Color indicates TCox10$^{-/-}$ vs WT or TCox10$^{-/-}$/AOX vs TCox10$^{-/-}$ comparisons. Pathways that with padj < 0.25 in either or both comparisons are shown.

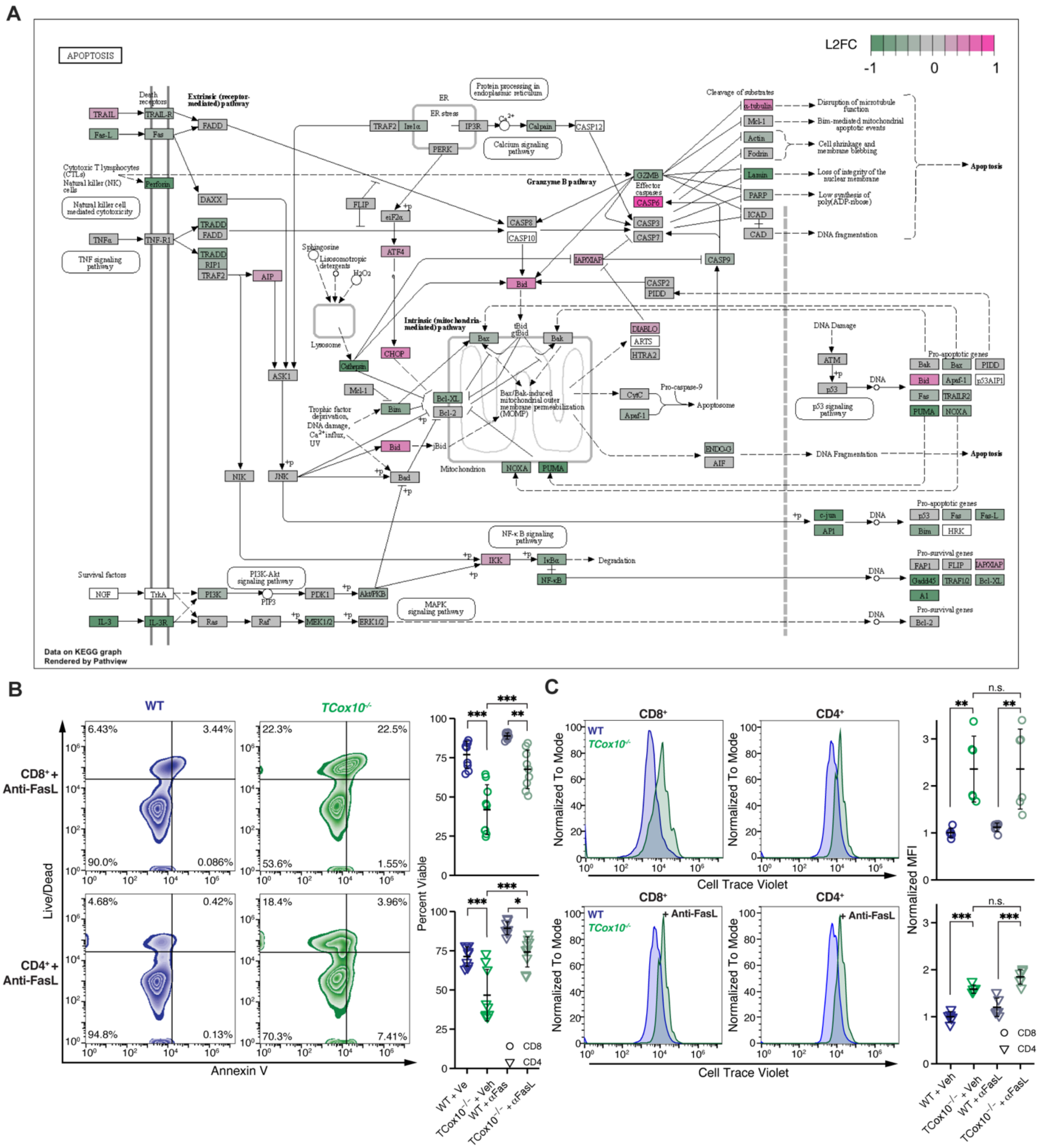


**Supplementary Fig. 3. Aberrant apoptosis in *TCox10$^{-/-}$* T cells is abrogated by *Aox* expression and anti-FasL treatment.** (A) KEGG apoptosis pathway with relative expression (log2 fold change) from *TCox10$^{-/-}$/Aox* and *TCox10$^{-/-}$* visualized. (B) Viability analysis in CD4$^+$ and CD8$^+$ WT and *TCox10$^{-/-}$* T cells using Live/Dead and Annexin V stains, following treatment with anti-FasL (CD178) blocking antibodies. Left, contour plot; right, quantification of percent viable cells. (C) Proliferation analysis of CD4$^+$ and CD8$^+$ WT and T cells using Cell Trace Violet. Left, representative density plot. Right, quantification of normalized mean fluorescence intensity (MFI). Data are representative of two independent experiments and an RNAseq experiment and indicate mean and standard deviation. (A) n = 3-5, (B-C) n = 6. * $p < 0.05$, ** $p < 0.01$, *** $p < 0.001$ by

one-way ANOVA and post-hoc Tukey HSD test. Negative controls are not included in statistical calculations.

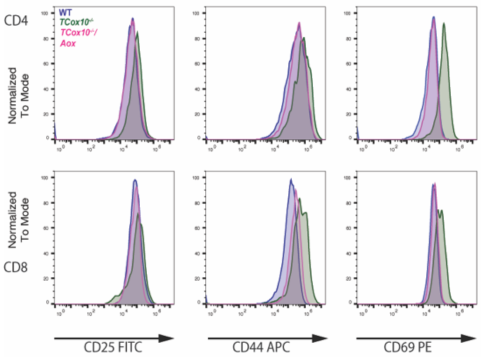


**Supplementary Fig. 4. Activation markers in T cells after 72 hours of stimulation.** Pan T cells were isolated by magnetic beads and stimulated with anti-CD3 and anti-CD28 for 72 hours in complete media , gated for live $CD4^+$ or $CD8^+$ cells. Cells were isolated by magnetic beads and stimulated for 72 hours with anti-CD3 and anti CD28.

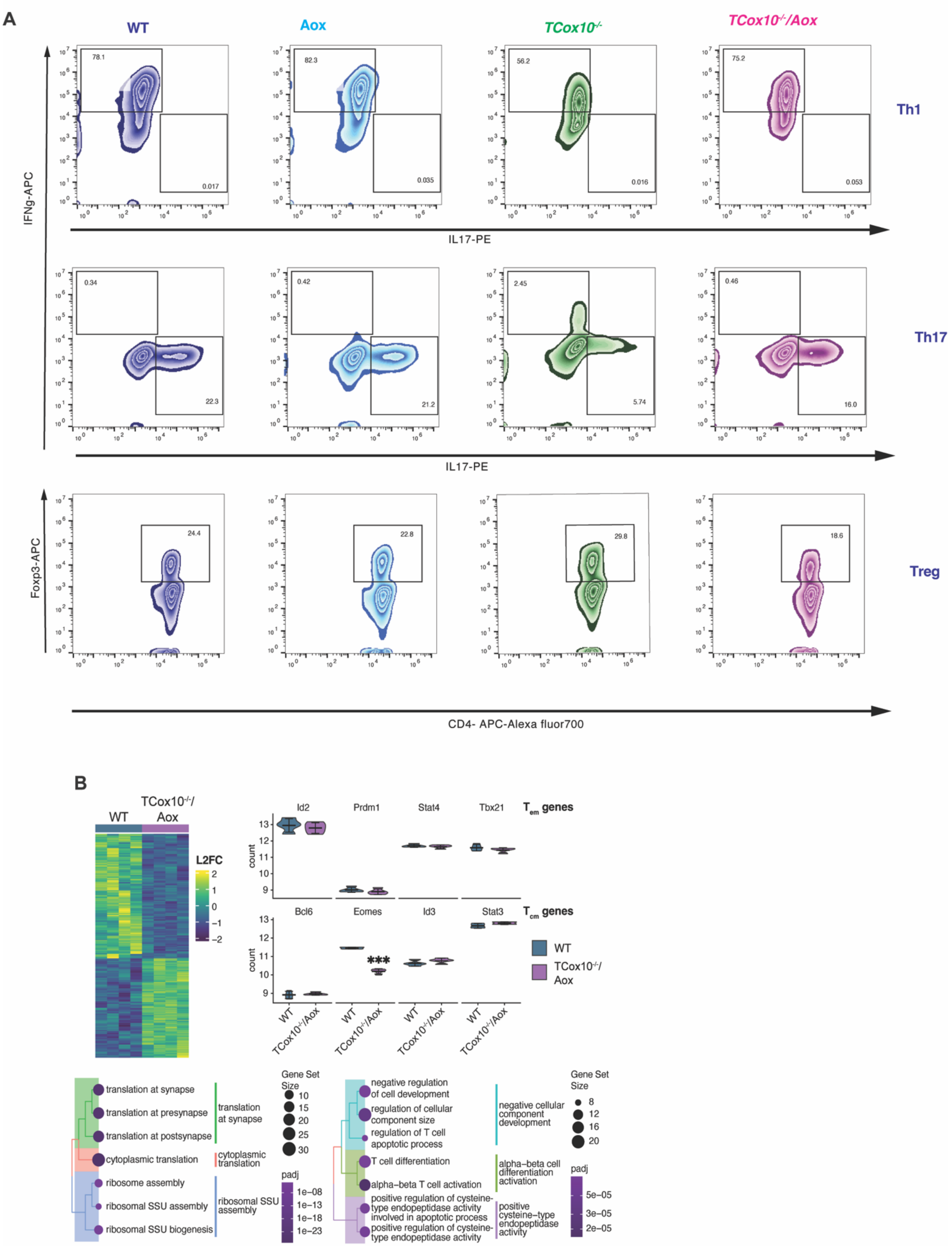


**Supplementary Fig. 5.** (A) Flow cytometry staining of Th1, Th17 and Treg cells. In brief, splenocytes are isolated from mouse spleen, sorted for naïve T cells (Tn) and activated with

subtype-specific factors to differentiate into Th1, Th17, or $T_{regs}$ as explained in Fig. 6D. After 3 days cells were stimulated with Tstim cocktail for 4 hours (for Th1 and Th17 conditions) and intracellular staining with $IFN_g$ and IL17 were evaluated by flow cytometry. Foxp3 staining was performed on non restimulated cells. (B) Differential expression between IL-15-differentiated WT and *TCox10*$^{-/-}$*/Aox* $T_{mem}$ cells. Top left, heatmap of significant differentially expressed genes. Top right, normalized expression of $T_{em}$ and $T_{cm}$ genes. Bottom left, ORA of significantly upregulated genes in *TCox10*$^{-/-}$*/Aox* Tmem cells. Bottom right, ORA of significantly downregulated genes in *TCox10*$^{-/-}$*/Aox* $T_{mem}$ cells.

**A**

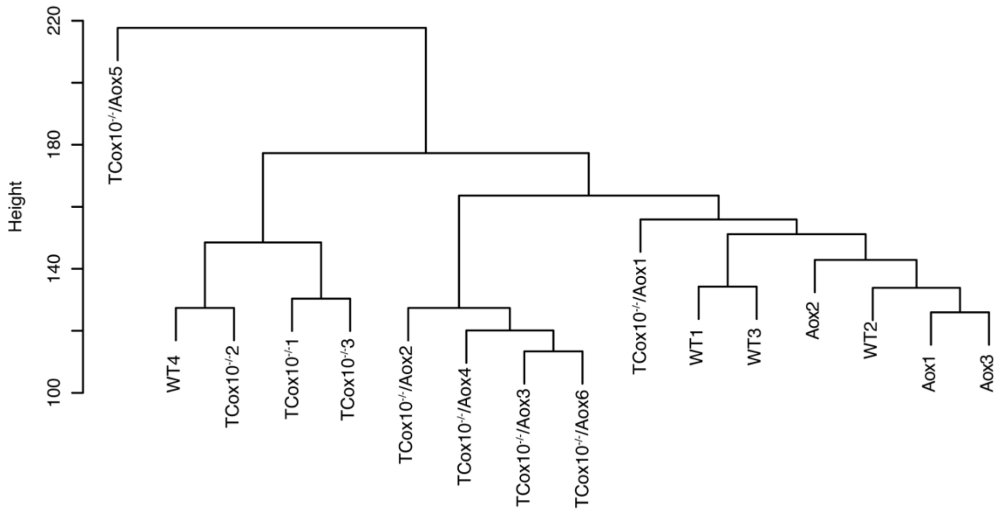


**B**

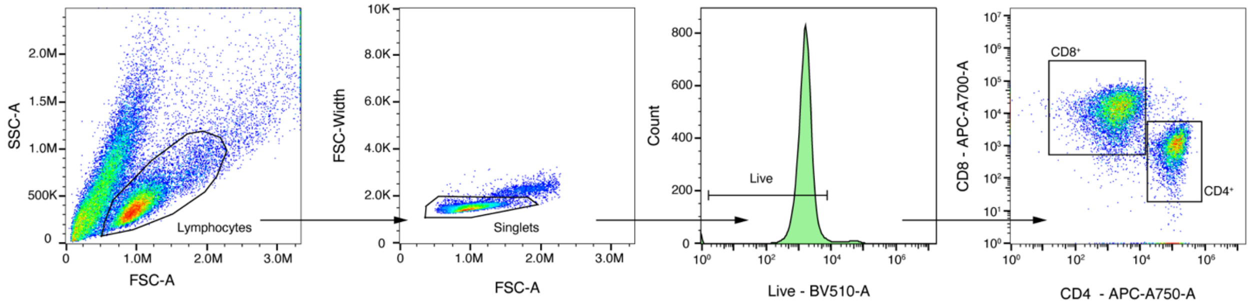


**Supplementary Fig. 6. Sample handling strategies.** (A) Hierarchical clustering revealed RNAseq sample *TCox10*$^{-/-}$*/Aox 5* partitioned separately from others; this sample was excluded from our analysis. (B) Representative gating strategy for $CD8^+$ and $CD4^+$ T cells in this manuscript.
