# Supplementary material for: Cytochrome c oxidase dependent respiration is essential for T cell activation, proliferation and memory formation
Source: Nat Commun. 2025 Dec 4;16:10898. doi: 10.1038/s41467-025-65910-w (PMC12678437; doi:10.1038/s41467-025-65910-w)
Supplement: Supplementary file 2 — Description of Additional Supplementary Files [file 41467_2025_65910_MOESM2_ESM.pdf]

Description of Additional Supplementary Files

Supplementary Data 1 (Excel file). Differential gene expression statistics underlying volcano plots. Source data for Figure 1 and Supplementary Data Fig 1. Differential expression analyses were performed for WT vs. *TCox10*$^{-/-}$, WT vs. *Aox*, WT vs. *TCox10*$^{-/-}$*/Aox*, and *TCox10*$^{-/-}$ vs. *TCox10*$^{-/-}$*/Aox* T cells. Reported values include gene name, base mean expression, log_2 fold change, test statistic, P value, and adjusted P value.

Supplementary Data 2 (Excel file). Overrepresentation analysis of gene co-expression modules. Table summarizes the results of overrepresentation analysis (ORA) performed on each gene module identified in the dataset for Figure 1. The analysis indicates the predominant biological functions and pathways associated with the modules. Reported values include ontology, description, BgRatio, P value, adjusted P value, Q value, gene ID, and counts. These data define the major functions represented in the gene sets underlying the modules.

Supplementary Data 3 (Excel file). Focused transcriptomic analysis of mitochondrial pathways in TCox10-/-/AOX T cells. Table contains the pathway-level transcriptomic results corresponding to Supplementary Data Fig. 2. Analyses were performed to determine whether *Aox* expression influences mitochondrial programs in *TCox10*$^{-/-}$ T cells. Reported values include gene ID and description, set size, enrichment score, normalized enrichment score (NES), P value, adjusted P value, Q value, rank, leading edge, core enrichment.

Supplementary Data 4 (Excel file). Transcriptomic analysis of memory T cell differentiation in *TCox10*$^{-/-}$*/Aox* cells. This table contains the RNAseq results corresponding to Supplementary Data Fig. 5B. Differential expression was assessed in *TCox10*$^{-/-}$*/Aox* memory T cells compared to WT controls. Reported values include gene name, base mean expression, log_2 fold change, test statistic, P value, and adjusted P value.
