# Supplementary material for: Cytochrome c oxidase dependent respiration is essential for T cell activation, proliferation and memory formation
Source: Nat Commun. 2025 Dec 4;16:10898. doi: 10.1038/s41467-025-65910-w (PMC12678437; doi:10.1038/s41467-025-65910-w)
Supplement: Supplementary file 7 — Reporting Summary [file 41467_2025_65910_MOESM7_ESM.pdf]

nature portfolio

Corresponding author(s): Peter J. McGuire

Last updated by author(s): Jul 18, 2025

Reporting Summary

Nature Portfolio wishes to improve the reproducibility of the work that we publish. This form provides structure for consistency and transparency in reporting. For further information on Nature Portfolio policies, see our Editorial Policies and the Editorial Policy Checklist.

Statistics

For all statistical analyses, confirm that the following items are present in the figure legend, table legend, main text, or Methods section.

n/a	Confirmed	
☐	☒	The exact sample size (*n*) for each experimental group/condition, given as a discrete number and unit of measurement
☐	☒	A statement on whether measurements were taken from distinct samples or whether the same sample was measured repeatedly
☐	☒	The statistical test(s) used AND whether they are one- or two-sided<br>*Only common tests should be described solely by name; describe more complex techniques in the Methods section.*
☐	☒	A description of all covariates tested
☐	☒	A description of any assumptions or corrections, such as tests of normality and adjustment for multiple comparisons
☐	☒	A full description of the statistical parameters including central tendency (e.g. means) or other basic estimates (e.g. regression coefficient) AND variation (e.g. standard deviation) or associated estimates of uncertainty (e.g. confidence intervals)
☐	☒	For null hypothesis testing, the test statistic (e.g. *F*, *t*, *r*) with confidence intervals, effect sizes, degrees of freedom and *P* value noted<br>*Give P values as exact values whenever suitable.*
☒	☐	For Bayesian analysis, information on the choice of priors and Markov chain Monte Carlo settings
☐	☒	For hierarchical and complex designs, identification of the appropriate level for tests and full reporting of outcomes
☒	☐	Estimates of effect sizes (e.g. Cohen's *d*, Pearson's *r*), indicating how they were calculated

Our web collection on statistics for biologists contains articles on many of the points above.

Software and code

Policy information about availability of computer code

Data collection	NA
Data analysis	Statistical significance was determined using GraphPad Prism v10 or R 4.3.2. All flow cytometry data were analyzed with FlowJo v10.

For manuscripts utilizing custom algorithms or software that are central to the research but not yet described in published literature, software must be made available to editors and reviewers. We strongly encourage code deposition in a community repository (e.g. GitHub). See the Nature Portfolio guidelines for submitting code & software for further information.

Data

Policy information about availability of data

All manuscripts must include a data availability statement. This statement should provide the following information, where applicable:

- Accession codes, unique identifiers, or web links for publicly available datasets
- A description of any restrictions on data availability
- For clinical datasets or third party data, please ensure that the statement adheres to our policy

Data is available upon request.

Research involving human participants, their data, or biological material

Policy information about studies with human participants or human data. See also policy information about sex, gender (identity/presentation), and sexual orientation and race, ethnicity and racism.

Reporting on sex and gender	NA
Reporting on race, ethnicity, or other socially relevant groupings	NA
Population characteristics	NA
Recruitment	NA
Ethics oversight	NA

Note that full information on the approval of the study protocol must also be provided in the manuscript.

Field-specific reporting

Please select the one below that is the best fit for your research. If you are not sure, read the appropriate sections before making your selection.

☐ Life sciences ☐ Behavioural & social sciences ☐ Ecological, evolutionary & environmental sciences

For a reference copy of the document with all sections, see nature.com/documents/nr-reporting-summary-flat.pdf

Life sciences study design

All studies must disclose on these points even when the disclosure is negative.

Sample size	The sample size was determined based on previous studies in the field, available resources, and the need to obtain preliminary results.
Data exclusions	Hieracrchical clustering was used to exclude a single sample in a single RNAseq analysis.Details are in Extended Data Fig. 4.
Replication	Experiments were repeated 3+ times where appropriate.
Randomization	Randomization of mice was not applied.
Blinding	NA

Behavioural & social sciences study design

All studies must disclose on these points even when the disclosure is negative.

Study description	NA
Research sample	NA
Sampling strategy	NA
Data collection	NA
Timing	NA
Data exclusions	NA
Non-participation	NA
Randomization	NA

Ecological, evolutionary & environmental sciences study design

All studies must disclose on these points even when the disclosure is negative.

Study description	NA
Research sample	NA

Sampling strategy	NA
Data collection	NA
Timing and spatial scale	NA
Data exclusions	NA
Reproducibility	NA
Randomization	NA
Blinding	NA

Did the study involve field work? ☐ Yes ☒ No

Reporting for specific materials, systems and methods

We require information from authors about some types of materials, experimental systems and methods used in many studies. Here, indicate whether each material, system or method listed is relevant to your study. If you are not sure if a list item applies to your research, read the appropriate section before selecting a response.

Materials & experimental systems

n/a	Involved in the study
☐	☒ Antibodies
☐	☒ Eukaryotic cell lines
☒	☐ Palaeontology and archaeology
☐	☒ Animals and other organisms
☒	☐ Clinical data
☒	☐ Dual use research of concern
☒	☐ Plants

Methods

n/a	Involved in the study
☒	☐ ChIP-seq
☐	☒ Flow cytometry
☒	☐ MRI-based neuroimaging

Antibodies

Antibodies used

CD44 PE clone IM7 12-0441-83 E01240-366 ebioscience
CD25 FITC 7D4 553072 9073690 BD bioscience
CD69 PE H1.2F3 12-0691-82 2009775 thermofisher
IFN APC XMG1.2 17-7311-82 2524225 thermofisher
CD4 AlexaFluor700 GK1.5 56-0041-82 2731885 thermofisher
CD8 AlexaFluor780 53-6.7 47-0081-82 2783679 thermofisher
Fas(CD95) PE 15A7 12-0951-81 E01394-1634 ebioscience
FasL(CD178) APC MFL3 17-5911-80 E13816-106 ebioscience
IL-17 PE eBio1787 12-7177-81 2674271 thermofisher
FOXP3 APC JFK-16s 17-5773-82 2555811 thermofisher
Granzyme PE 3G8.5 149704 B208581 biolegend
Ly6C efluor450 HK1.4 48-5932-82 2095541 ebioscience
CD62L efluor450 MEL-14 48-0621-82 2191857 ebioscience
B220 Alexafluor780 RAS-6B2 47-0452-82 2703742 thermofisher
Perforin APC S16009A 154304 B274826 Biolegend

Validation

Negative controls were used to define cell populations.

Eukaryotic cell lines

Policy information about cell lines and Sex and Gender in Research

Cell line source(s)	EL-4 (ATCC)
Authentication	No formal authentication of the cell lines was carried out.
Mycoplasma contamination	Cell lines were tested for mycoplasma using PCR. The results were negative.
Commonly misidentified lines (See ICLAC register)	*Name any commonly misidentified cell lines used in the study and provide a rationale for their use.*

Animals and other research organisms

Policy information about studies involving animals; ARRIVE guidelines recommended for reporting animal research, and Sex and Gender in Research

Laboratory animals	B6-Cox10tm1Ctm/J<br>B6.CgTg(Cd4-cre)1Cwi/BfluJ<br>B6Tg(CAG-Aox)Ht
Wild animals	NA
Reporting on sex	Sex was not a variable in the experiments conducted. No functional differences were observed between male and female mice.
Field-collected samples	NA
Ethics oversight	NHGRI Animal Care and Use Committee

Note that full information on the approval of the study protocol must also be provided in the manuscript.

Plants

Seed stocks	NA
Novel plant genotypes	NA
Authentication	NA

Flow Cytometry

Plots

Confirm that:

- [x] The axis labels state the marker and fluorochrome used (e.g. CD4-FITC).
- [x] The axis scales are clearly visible. Include numbers along axes only for bottom left plot of group (a 'group' is an analysis of identical markers).
- [x] All plots are contour plots with outliers or pseudocolor plots.
- [x] A numerical value for number of cells or percentage (with statistics) is provided.

Methodology

Sample preparation	Cells obtained from cell cultures or extracted from the spleen using isolation kits.
Instrument	Cytoflex LX and Cytoflex S
Software	FLowJo version 10.0
Cell population abundance	All cell populations analyzed are quantified as indicated.
Gating strategy	The cells were selected by FSC and SSC parameters and subsequently by viability.

- [x] Tick this box to confirm that a figure exemplifying the gating strategy is provided in the Supplementary Information.
